# Relationship of cigarette smoking and hearing loss in workers exposed to occupational noise

**DOI:** 10.1186/2052-4374-25-8

**Published:** 2013-07-03

**Authors:** Joo Hyun Sung, Chang Sun Sim, Choong-Ryeol Lee, Cheol-In Yoo, Hun Lee, Yangho Kim, Jiho Lee

**Affiliations:** 1 Department of Occupational and Environmental Medicine, Ulsan University Hospital, University of Ulsan College of Medicine, 290-3, Jeonha-dong, Dong-gu, Ulsan 682-714, South Korea

**Keywords:** Hearing loss, Occupational noise, Smoking

## Abstract

**Objectives:**

To investigate the effects of smoking on hearing loss among workers exposed to occupational noise.

**Methods:**

From the results of a special workers health examination performed in 2011, we enrolled 8,543 subjects exposed to occupational noise and reviewed the findings. Using self-reported questionnaires and health examination results, we collected data on age, smoking status, disease status, height, weight, and biochemistry and pure tone audiometry findings. We divided the workers into 3 groups according to smoking status (non-smoker, ex-smoker, current smoker). Current smokers (n = 3,593) were divided into 4 groups according to smoking amount (0.05–9.9, 10–19.9, 20–29.9, ≥30 pack-years). We analyzed the data to compare hearing thresholds between smoking statuses using analysis of covariance (ANCOVA) after controlling for confounder effects.

**Results:**

According to ANCOVA, the hearing thresholds of current smokers at 2 k, 3 k, and 4 kHz were significantly higher than that of the other groups. Multiple logistic regression for smoking status (reference: non-smokers) showed that the adjusted odds ratios of current smokers were 1.291 (95% confidence interval [CI]: 1.055–1.580), 1.180 (95% CI: 1.007–1.383), 1.295 (95% CI: 1.125–1.491), and 1.321 (95% CI: 1.157–1.507) at 1 k, 2 k, 3 k, and 4 kHz, respectively. Based on smoking amount, the adjusted odds ratios were 1.562 (95% CI: 1.013–2.408) and 1.643 (95% CI: 1.023–2.640) for the 10–19.9 and ≥30 pack-years group, respectively, at 1 kHz (reference: 0.05–9.9 pack-years). At 2 kHz, the adjusted odds ratios were increased statistically significantly with smoking amount for all groups. At all frequencies tested, the hearing thresholds of noise-exposed workers were significantly influenced by current smoking, in particular, the increase of hearing loss at low frequencies according to smoking amount was more prevalent.

**Conclusions:**

Current smoking significantly influenced hearing loss at all frequencies in workers exposed to occupational noise, and heavier smoking influenced low-frequency hearing loss more greatly. There was a dose–response relationship between smoking amount and low-frequency hearing thresholds; however, this was not observed for high-frequency hearing thresholds. Therefore, well-designed prospective studies are needed to clarify the effects of smoking on the degree of hearing loss.

## Introduction

Noise is the most common detrimental physical factor in the working environment. According to the distribution of occupational diseases in Korea, noise-induced hearing loss was reported in 55.5% of total patients with occupational diseases in 1991, being the most prevalent occupational disease. Since then, its incidence has continually increased. According to 2010 report by the Ministry of Employment and Labor [1], workers with noise-induced hearing loss (D1) account for 93.3% (5,065/5,426) of total workers with occupational diseases, and those with probable noise-induced hearing loss (C1) account for 92.3% (108,213/117,270) of total workers with probable occupational diseases. In addition, of 1,924,305 workers who are supposed to undergo a special health examination, 516,828 (26.9%) are exposed to detrimental noise before undergoing a noise-specific health examination. Therefore, this will also cause continual problems. According to the National Institute for Occupational Safety and Health (NIOSH) reports, 14% of workers in the United States work in an environment where the noise level exceeds 90 dBA [2]. The social and medical expenditure for the treatment, rehabilitation, and compensation of workers with noise-induced hearing loss are enormous [3]. Moreover, noise-induced hearing loss decreases the quality of life of such workers and causes problems such as social disconnection, depression, and increased risk of being involved in an accident. In this regard, noise-induced hearing loss is a serious problem [2,4]. Therefore, to prevent these problems from occurring, methods for reducing noise-induced hearing loss should be developed as quickly as possible. The currently available effective methods for preventing the occurrence of noise-induced hearing loss include the use of hearing protection devices [5]. The identification and management of factors that may affect noise-induced hearing loss would be more effective as well. Further, this will also be helpful for improving the quality of life of workers.

Noise-induced hearing loss is also sensorineural hearing loss. It is known to occur when individuals are exposed to noise that exceeds 85 dBA. However, there is great discrepancy in the sensitivity for noise-induced hearing loss between individuals. That is to say, some people may tolerate loud noise, but others in the same environment may experience rapid loss of hearing [6]. The factors known to be associated with sensitivity to noise-induced hearing loss are sex, age, smoking, cardiovascular factors (body mass index [BMI], blood pressure, total cholesterol, triglyceride, high-density lipoprotein [HDL] cholesterol, and fasting blood sugar) and factors associated with blood viscosity (hemoglobin, hematocrit, and red blood cell [RBC] count) [7-9].

Smoking an adjustable risk factor and is a cause of the most life-threatening chronic diseases and premature death. The rate of smoking in adult men was highest (79.3%) in 1980 and decreased thereafter to 42.0% in 2007. However, the number of female and adolescent smokers has tended to increase as compared with the past. Thus, smoking remains a problem for which social coordination is required. Kim et al. [10] reported that smoking incurs such a great social loss that social expenditure due to smoking, except indirect smoking, exceeded KRW 4,000 billion in Korea in 2006. Furthermore, smoking is a cause of various cancers, respiratory diseases, and cardiovascular diseases. Recent studies have shown that it also has a detrimental effect on hearing function and the endocrine system [11].

To date, studies that examined the relationship between smoking and noise-induced hearing loss have reported that the incidence of noise-induced hearing loss is significantly higher in smokers [12-14] or that there is a synergistic effect between the 2 factors [15,16]. Yet, other studies have reported that there is no significant correlation between the 2 factors [17]. Recent studies have shown that smoking itself is not a cause of sensorineural hearing loss, but exerts a synergistic effect when individuals are exposed to smoking together with noise [2,18]. In addition, some studies have shown that there is a dose–response relationship between the amount of smoking and noise-induced hearing loss [19]. In Korea, however, few studies have recently examined the relationship between smoking and noise-induced hearing loss as compared with other countries, where such relevant studies are actively conducted. Therefore, there is no evidence for implementing healthcare policies such as a no-smoking workplace together with those for the prevention of noise-induced hearing loss.

We therefore conducted this study to examine the effects of smoking on the development of noise-induced hearing loss. We attempted to provide baseline evidence for reduction of the occurrence of noise-induced hearing loss by government and company policies regarding a no-smoking workplace, prevention of the development of various diseases due to smoking, improvement of the quality of life of workers, and reduction of social expenditure. In the current study, we evaluated the effects of smoking status and smoking amount on hearing loss in workers exposed to noise in the workplace. Moreover, we attempted to examine the effects of smoking in this setting and the factors that may have affected sensitivity were adjusted.

## Materials

### Study population

**Figure 1:**
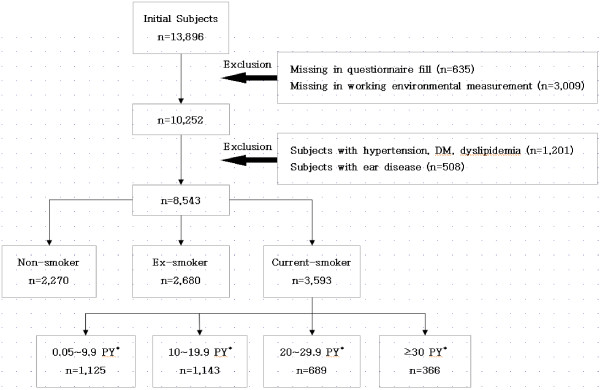
Selection of subjects. ^*^ PY: pack-years.

The current study was conducted on 13,896 workers at a local shipyard who were aged between 20 and 62 years and who underwent a noise-specific health examination during a 1-year period from March 1, 2010, to February 28, 2011. We excluded 635 workers who did not respond to the questionnaire survey; 3,009 from whom we did not receive the result of the measurement of workplace environment noise; 1,201 with diseases known to have a significant effect on hearing function, e.g., hypertension, diabetes mellitus, and hyperlipidemia; and 508 who had a past history of ear diseases on otoscopy and interview. We therefore enrolled 8,543 workers in the current study, dividing them into non-smoker, ex-smoker, and current smoker groups according to their smoking status. We also divided the current smoker group into 4 subgroups according to smoking amount (pack-years): 0.05–9.9, 10–19.9, 20–29.9, and ≥30 (Figure 1).

All analyses were performed mainly for the right ear because the mean hearing threshold was relatively lower. Based on frequency, we divided the subjects into the normal hearing function group and the hearing loss group. Subjects were assigned to the hearing loss group when the hearing threshold exceeded 30 dBHL at 1 k Hz. Subjects were also assigned to the hearing loss group when their hearing thresholds exceeded 30, 40, and 40 dBHL at 2 k, 3 k, and 4 kHz, respectively. All criteria were indications for a secondary test for the noise-specific health examination [20].

### Study methods

#### Questionnaire study

Using a self-reporting questionnaire, we examined age, period of noise exposure, past medical history, current illness, smoking status, and past and current history of ear diseases. Smoking status was evaluated according to the criteria for health examination (Korean Ministry of Health and Welfare Notification No. 2012–69). Thus, based on the questionnaire survey, subjects with no smoking history and those with a lifetime smoking history of <100 cigarettes were assigned to the non-smoker group. Subjects with past smoking history but who did not smoke cigarettes currently and those with a lifetime smoking history of ≥100 cigarettes were assigned to the ex-smoker group. Subjects who smoked cigarettes currently were assigned to the current smoker group [21].

The smoking amount (pack-years) was calculated by dividing the daily number of cigarettes smoked by 20, and then multiplying it with the number of years of smoking. In addition, the subjects in the current smoker group were divided into 4 subgroups according to smoking amount (pack-years): 0.05–9.9, 10–19.9, 20–29.9, and ≥30.

#### Measurement of noise levels

Noise was measured according to the criteria for working environment measurement (Ministry of Employment and Labor Notification No. 2009–78, dated February 14, 2009). We reviewed the results of the measurement of workplace environment noise using devices that measured the amount of noise exposure (2 noise-logging dosimeters [M-27 and M-28, QUEST, USA], an audio dosimeter [MK-3, AMETEK, USA], a noise dose badge [CR100, CIRRUS, England]) from January 2010 to June 2010. The mean noise level of each department to which the workers belonged was assumed as the noise level of the worker.

#### Physical examination and serum biochemistry

The height, weight, and systolic and diastolic blood pressure were measured. Fasting blood sugar, total cholesterol, triglyceride, HDL cholesterol, hemoglobin, hematocrit, RBC count, and carboxyhemoglobin were also measured. The BMI was calculated by dividing the weight (kg) by the squared height in meters. Blood pressure was calculated according to the following formula:

$$\begin{array}{l} \mathrm{Mean}\;\mathrm{arterial}\;\mathrm{pressure}\\ \;=\left(\frac{\mathrm{systolic}\;\mathrm{pressure}+\mathrm{diastolic}\;\mathrm{pressure}\times 2}{3}\right) \end{array}$$

The mean arterial pressure reflects the mean effective pressure [8].

#### Audiometry

To measure the hearing thresholds in workers exposed to occupational noise, experienced testers measured the threshold of air-conducted pure tone audiometry at 1 k, 2 k, 3 k, and 4 kHz using the modified Hughson-Westlake procedure, a standardized method of measurement. To do this, we used an Interacoustic AC40 audiometer (Denmark) and TDH39-P headphones, which can measure frequencies of 0.25–16 KHz. The noise around the booth was appropriate for the American National Standards Institute (ANSI) S3.1-1999 criteria. In addition, an audiometric device was adjusted according to the guidelines for audiometry of the Korean Occupational Safety and Health Agency [22,23].

#### Statistics

Subjects were assigned to non-smoker, ex-smoker, and current smoker groups. We compared age, number of years working, noise level, various physical parameters, biochemistry results, pure tone audiometry results, and the mean arterial pressure with 1-way analysis of variance. Data were analyzed to compare hearing thresholds between smoking statuses and between smoking amounts using analysis of covariance (ANCOVA) and multiple logistic regression after controlling for confounder effects. Age, number of years working, noise level, cardiovascular factors (BMI, mean arterial pressure, fasting blood sugar, total cholesterol, triglyceride, and HDL cholesterol), and factors associated with blood viscosity (hemoglobin) served as covariates. Statistical analysis was performed using SPSS 19.0 (IBM SPSS Inc., Chicago, IL, USA). P-value < 0.05 was considered statistically significant.

## Results

### Subject characteristics

We compared the baseline characteristics between the 1,709 excluded subjects with diseases affecting the hearing threshold or in whom the presence of such diseases was confirmed upon otoscopy or interview and the 8,543 enrolled subjects. Age, working period, mean arterial pressure, and fasting blood sugar were significantly higher in the excluded subjects. Moreover, the hearing thresholds of the excluded subjects were significantly higher within the overall range of frequencies (data not shown).

**Figure 2:**
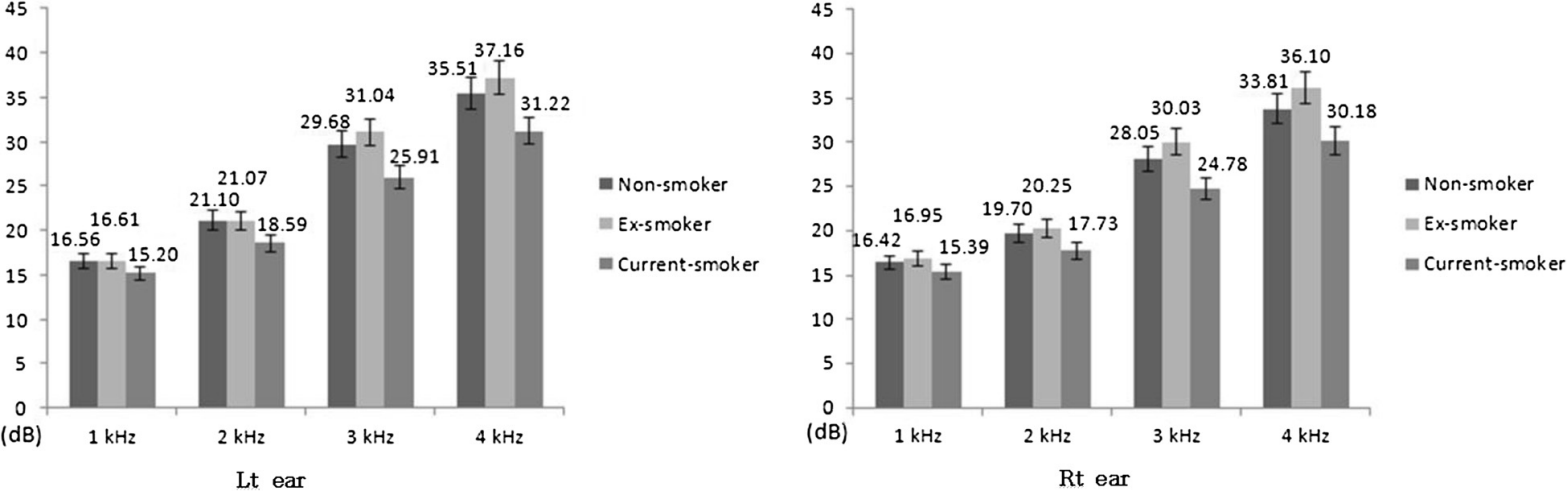
Hearing thresholds of the subjects according to smoking status. ^*^ Bar: the mean of hearing threshold and error range (±5%) of the both ear at all frequency.

We enrolled 8,543 subjects in the current study. Of these, 2,270, 2,680, and 3,593 subjects were assigned to the non-smoker, ex-smoker, and current smoker group, respectively. Results are expressed as mean ± SD (standard deviation). The mean age was 46.3 ± 9.4 years, the mean number of years working was 22.1 ± 10 years, and the mean noise level was 86.7 ± 3.0 dB. Age and working period, and noise level were significantly low in the current smoker and ex-smoker groups, respectively. The BMI was low in the non-smoker group. The mean arterial pressure and fasting blood sugar were low in the current smoker group. Lipid profile analysis showed that total cholesterol and triglyceride, and HDL cholesterol were low in the non-smoker and current smoker groups, respectively. These results revealed a significant difference between the 3 groups. Hematocrit and carboxyhemoglobin were high in the current smoker group. RBC counts were low in the ex-smoker group.. The pure tone audiometry results showed that the mean hearing threshold of the current smoker group was significantly low within the overall range of frequencies tested (Table 1, Figure 2).

**Table 1:** Comparisons of general characteristics on subjects according to smoking status

	**Non**-smoker^* ^**(n = 2,270)**	Ex-smoker^* ^**(n =** **2,680)**	Current smoker^* ^**(n =** **3,593)**	**Total (n =** **8,543)**	***p *** **value**^**†**^	**Post-hoc comparison**
Age (years)	47.5 ± 9.4	48.9 ± 8.0	43.5 ± 9.7	46.3 ± 9.4	<0.001	c < a < b
Working period (years)	23.1 ± 10.2	24.4 ± 9.2	19.8 ± 10.0	22.1 ± 10.0	<0.001	c < a < b
Noise level (decibel)	86.8 ± 3.0	86.5 ± 2.9	86.8 ± 3.1	86.7 ± 3.0	0.001	b < a,c
Body mass index (kg/m^2^)	23.4 ± 2.3	23.5 ± 2.3	23.4 ± 2.5	23.4 ± 2.4	0.013	a < b
Mean arterial pressure (mmHg)	93.2 ± 8.5	93.3 ± 8.5	91.9 ± 8.5	92.7 ± 8.5	<0.001	c < a,b
Fasting blood sugar (mg/㎗)	98.2 ± 12.0	98.9 ± 12.8	97.4 ± 12.8	98.1 ± 12.6	<0.001	c < b
Total cholesterol (mg/㎗)	188.9 ± 31.7	191.4 ± 31.9	190.2 ± 32.3	190.2 ± 32.0	0.023	a < b
Triglyceride (mg/㎗)	115.7 ± 71.5	123.8 ± 88.2	141.9 ± 105.8	129.3 ± 92.9	<0.001	a < c
HDL cholesterol (mg/㎗)	53.5 ± 13.1	53.1 ± 12.9	51.4 ± 12.7	52.5 ± 12.9	<0.001	c < a,b
Hemoglobin (g/㎗)	14.9 ± 0.9	14.9 ± 0.9	15.1 ± 0.9	15.0 ± 0.9	<0.001	a,b < c
Hematocrit (%)	43.4 ± 2.6	43.1 ± 2.6	43.9 ± 2.7	43.5 ± 2.7	<0.001	a,b < c
	(n = 735)	(n = 800)	(n = 1,227)	(n = 2,762)		
Red blood cell count (10^4^/㎣)	468.9 ± 32.8	465.5 ± 33.4	468.8 ± 34.2	467.9 ± 33.6	0.020	b < a,c
	(n = 1,084)	(n = 1,129)	(n = 1,673)	(n = 3,886)		
Carboxyhemoglobin (%)	0.58 ± 0.75	0.52 ± 0.70	2.26 ± 1.83	1.29 ± 1.57	<0.001	a,b < c
	(n = 627)	(n = 679)	(n = 1,007)	(n = 2,313)		
Pure tone audiometry of right ear (dBHL)						
1 kHz	16.4 ± 11.9	16.9 ± 12.2	15.4 ± 10.5	16.2 ± 11.4	<0.001	c < a,b
2 kHz	19.7 ± 15.2	20.3 ± 15.3	17.7 ± 13.8	19.0 ± 14.7	<0.001	c < a,b
3 kHz	28.1 ± 20.3	30.0 ± 20.8	24.8 ± 19.4	27.3 ± 20.2	<0.001	c < a < b
4 kHz	33.8 ± 22.0	36.1 ± 22.4	30.2 ± 21.7	33.0 ± 22.1	<0.001	c < a < b

mean ± standard deviation.

^*^ a: Non-smoker, b: Ex-smoker, c: Current smoker.

^†^*p*-value was calculated by ANOVA.

### Correlation between smoking status and hearing loss

**Figure 3:**
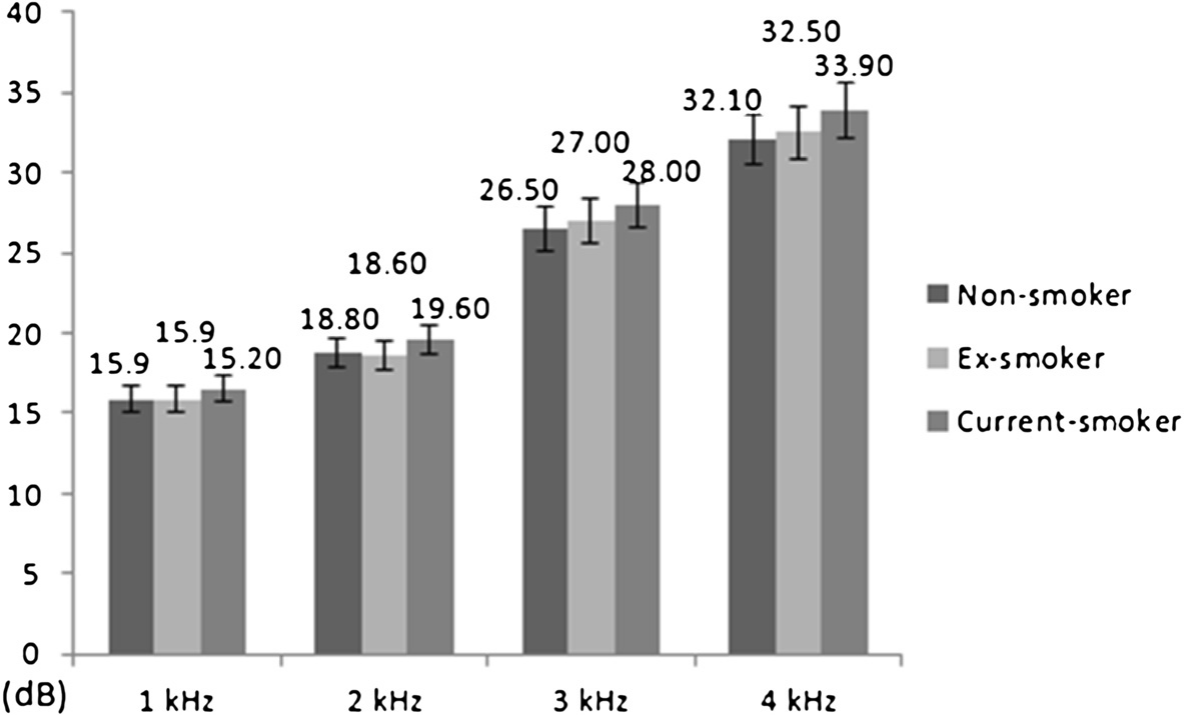
Hearing thresholds of the subjects according to smoking status after adjusting for confounding variables. ^*^ Bar: the mean of hearing threshold and error range (±5%) of the both ear at all frequency. ^†^ Confounding variables: age, working period, noise level, body mass index, mean arterial pressure, fasting blood glucose, total cholesterol, triglyceride, HDL cholesterol, hemoglobin.

**Figure 4:**
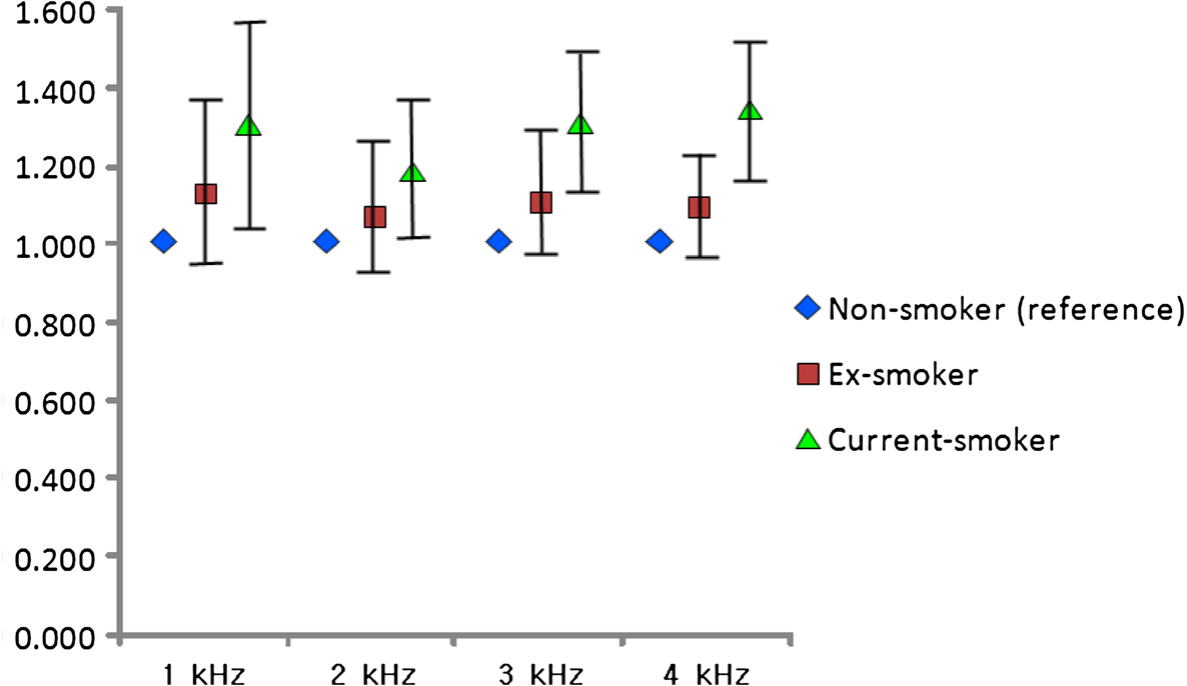
Adjusted odds ratio of hearing loss by frequencies according to smoking status. ^*^ Dot: odds ratio (adjusted for age, working period, noise level, body mass index, mean arterial pressure, fasting blood glucose, total cholesterol, triglyceride, HDL cholesterol, hemoglobin), bar: 95% confidence interval according to smoking status.

We analyzed the data with ANCOVA after controlling the effects of covariates such as age, working period, noise level, BMI, mean arterial pressure, fasting blood sugar, total cholesterol, triglyceride, HDL cholesterol, and hemoglobin. Pure tone audiometry revealed no significant correlation between the hearing threshold at 1 k Hz and smoking status. At 2 k, 3 k, and 4 kHz, however, the hearing threshold in the current smoker group was significantly high (Table 2, Figure 3). Multiple logistic regression for smoking status (reference: non-smokers) showed that the adjusted odds ratios of current smokers were 1.291 (95% confidence interval [CI]: 1.055–1.580), 1.180 (95% CI: 1.007–1.383), 1.295 (95% CI: 1.125–1.491), and 1.321 (95% CI: 1.157–1.507) at 1 k, 2 k, 3 k, and 4 kHz, respectively (Figure 4).

**Table 2:** Comparisons of pure tone audiometry threshold according to smoking status after adjusting for confounding variables

**Frequency**	**Non**-smoker^* ^**(n =** **2,270)**	Ex-smoker^* ^**(n =** **2,680)**	Current smoker^* ^**(n =** **3,593)**	***p *** **value**^**†**^	**Post-hoc comparison**
1 kHz	15.9 (15.47–16.37)	15.9 (15.51–16.34)	16.5 (16.10–16.83)	0.098	-
2 kHz	18.8 (18.24–19.35)	18.6 (18.04–19.07)	19.6 (19.11–20.01)	0.012	a,b < c
3 kHz	26.5 (25.81–27.22)	27.0 (26.36–27.67)	28.0 (27.43–28.58)	0.005	a,b < c
4 kHz	32.1 (31.30–32.80)	32.5 (31.84–33.24)	33.9 (33.34–34.55)	<0.001	a,b < c

mean (95% confidence interval), dBHL.

^*^ a: Non-smoker, b: Ex-smoker, c: Current smoker.

^†^*p*-value was calculated by ANCOVA and adjusted for age, working period, noise level, body mass index, mean arterial pressure, fasting blood glucose, total cholesterol, triglyceride, HDL cholesterol, hemoglobin.

### Correlation between smoking amount and hearing loss

The current smoker group was divided into 4 subgroups according to the amount of smoking (pack-years): 0.05–9.9, 10–19.9, 20–29.9, and ≥30. The criteria for determining hearing loss were the same as before. We analyzed the data using the same method. The hearing threshold at 1 k, 2 k, and 3 kHz was significantly high in the ≥30 pack-years group (Table 3). Multiple logistic regression for smoking amount (reference: 0.05–9.9 pack-years group) showed that at 1 kHz, the adjusted odds ratio was 1.562 (95% CI: 1.013–2.408) and 1.643 (95% CI: 1.023–2.640) for the 10–19.9 and ≥30 pack-years group, respectively. There was no significant difference between the 0.05–9.9 pack-years group and the 20–29.9 pack-years group. At 2 kHz, the adjusted odds ratio was 1.420 (95% CI: 1.014–1.988), 1.673 (95% CI: 1.179–2.374), and 1.660 (95% CI: 1.143–2.411) for the 10–19.9, 20–29.9, and ≥30 pack-years group, respectively. At 3 k and 4 kHz, however, there was no significant difference between smoking amounts. We also analyzed the trend at each frequency tested, but this also showed no significant difference (Table 4).

**Table 3:** Comparisons of pure tone audiometry threshold according to the smoking amount in current smoker

**Frequency**	**Smoking amount (pack-years)**				***p *** **value**^**†**^	**Post-hoc comparison**
	0.05–9.9^* ^**(n =** **1,125)**	10–19.9^* ^**(n =** **1,413)**	20–29.9^* ^**(n =** **689)**	≥30^* ^**(n =** **366)**		
1 kHz	15.2 (0.3)	15.2 (0.3)	15.3 (0.4)	16.9 (0.6)	0.030	a,b,c < d
2 kHz	17.5 (0.4)	17.2 (0.3)	18.1 (0.5)	20.1 (0.7)	0.002	a,b,c < d
3 kHz	25.1 (0.5)	23.7 (0.4)	25.2 (0.6)	27.2 (0.9)	0.001	b < a,c < d
4 kHz	30.5 (0.6)	29.3 (0.5)	30.8 (0.7)	31.5 (0.9)	0.075	-

mean (standard error).

^*^a: 0.05–9.9, b: 10–19.9, c: 20–29.9, d: ≥30 pack-years.

^†^*p*-value was calculated by ANCOVA and adjusted for age, working period, noise level, body mass index, mean arterial pressure, fasting blood glucose, total cholesterol, triglyceride, HDL cholesterol, hemoglobin.

**Table 4:** Adjusted odds ratio of hearing loss by frequencies according to the smoking amount in current smoker

	1 kHz^*^			2 kHz^*^			3 kHz^*^			4 kHz^*^		
	**N**	**OR**^**†**^	**95% CI**	**N**	**OR**^**†**^	**95% CI**	**N**	**OR**^**†**^	**95% CI**	**N**	**OR**^**†**^	**95% CI**
Smoking amount (pack-years)												
0.05-9.9	1,125	1.000		1,125	1.000		1,125	1.000		1,125	1.000	
10-19.9	1,413	1.562	1.013–2.408	1,413	1.420	1.014–1.988	1,413	1.063	0.811–1.393	1,413	1.034	0.815–1.312
20-29.9	689	1.557	0.990–2.450	689	1.673	1.179–2.374	689	1.198	0.898–1.599	689	1.228	0.945–1.595
≥30	366	1.643	1.023–2.640	366	1.660	1.143–2.411	366	1.264	0.918–1.742	366	1.060	0.779–1.442
*p* for trend		0.263			0.197			0.314			0.387	

^*^ 1 k, 2 kHz: threshold ≥ 30 dBHL included hearing loss group, 3 k, 4 kHz: threshold ≥ 40 dBHL included hearing loss group.

^†^ Adjusted for age, working period, noise level, body mass index, mean arterial pressure, fasting blood glucose, total cholesterol, triglyceride, HDL cholesterol, hemoglobin.

## Discussion

Several factors are known to affect noise-induced hearing loss. These include age, working period, noise level, cardiovascular factors, and factors associated with blood viscosity. We analyzed the data considering these factors and determined that the hearing threshold was highest within the overall range of frequencies tested other than 1 kHz on pure tone audiometry in the current smoker group. In addition, multiple logistic regression analysis revealed a significant difference within the overall range of frequencies tested in the current smoker group. In the ex-smoker group, however, there was no significant difference. Therefore, as compared with the non-smoker group, the current smoker group is at higher risk of developing hearing loss. It is also probable that current smokers with higher hearing thresholds in the frequency range ≥1 kHz would be more vulnerable to developing hearing loss. In the ex-smoker group, however, there was no statistical significance. It can therefore be inferred that current smoking, rather than past smoking history, has a greater effect on hearing function.

To date, it has been reported that the mechanisms by which noise-induced hearing loss occurs include 1) distortion and loss of the normal structure due to damage to the stereocilia after exposure to noise, and 2) damage to hair cells of the organ of Corti due to DNA damage (due to increased levels of toxic free radicals and reactive oxygen species generated during exposure to noise), degradation of lipid and protein, and acceleration of apoptosis [6,18]. The mechanisms by which smoking affects the auditory organ include 1) direct ototoxicity of nicotine, and 2) cochlear ischemia due to increased levels of carboxyhemoglobin, vasoconstriction, and increased blood viscosity due to smoking [24-28]. In addition, the cochlea is characterized by blood supply from a single blood vessel and a lack of collateral circulation. It is also characterized by very high metabolic activity of the hair cells. It is therefore known to be particularly vulnerable to ischemic injury. This has also been demonstrated in experimental studies [29,30]. Therefore, current smokers working in noisy workplaces would be more vulnerable to hearing loss as compared with non-smokers due to the complex involvement of these mechanisms.

The current study was conducted on workers who underwent a noise-specific health examination at a university hospital during a 1-year period. Therefore, we could not measure the serum levels of carboxyhemoglobin in all of the workers. Following analysis of the measurement in some workers, however, we determined that serum carboxyhemoglobin was significantly higher in the current smoker group. From the same perspective, it can also be inferred that the serum levels of carboxyhemoglobin would be high in other workers who were current smokers, and that this might cause hearing loss. Furthermore, it has been reported that cochlear damage may occur when there is an increase in the factors associated with blood viscosity, such as RBC count [24,31], white blood cell count, and increased hemoglobin and hematocrit [32,33]. RBC counts and hematocrit were not measured in all of the workers for the same reason carboxyhemoglobin was not measured. In the current study, RBC counts were not significantly high in the current smokers. However, hemoglobin and hematocrit were significantly high in this group. Therefore, it can be inferred that the increased blood viscosity due to increased hemoglobin and hematocrit causes ischemic damage to the cochlea. Thus, this may cause hearing loss. In the current study, total cholesterol was not significantly high in the current smokers. However, their triglyceride and HDL cholesterol levels were significantly high and low, respectively. Smoking may cause derangement of the serum lipid metabolism [11,34], lowering the serum levels of total and HDL cholesterol and increasing those of triglyceride [33]. We could not completely adjust all the effects on smoking and cholesterol because the study was conducted using a cross-sectional design on workers at a local shipyard. Therefore, our results should be interpreted only in a limited scope. Nevertheless, smoking may cause alterations in the serum lipid profile and thereby promote atherosclerotic progression of the cochlear vessels. Thus, this may cause hearing loss.

To summarize, if there is any chance that the effects of smoking-induced carboxyhemoglobin, ischemic damage due to increased blood viscosity, and the progression of atherosclerosis due to derangement of the serum lipid metabolism are the mechanisms by which noise-induced hearing loss occurs, the severity of hearing loss would be greater. Ahn et al. [18] conducted an animal experimental study using rats, reporting that there would be a delay in recovery from the temporary threshold shift if there were a chance that the effects of smoking were added to that of noise exposure. Mohammadi et al. [2] also reported that there would be a synergistic effect between noise exposure and smoking. Our results also showed that the degree of hearing loss was significantly high in the smokers who were exposed to noise. Considering our results and the theoretical background of previous studies, it can be inferred that smoking would have a detrimental effect on the hearing function of workers who are exposed to noise.

Previous studies have shown that there is variability in the range of frequencies in which smoking has an effect on hearing function. Specifically, it has previously been shown that smoking caused hearing loss at higher frequencies in the absence of exposure to noise [35,36]. However, other reports have stated that it caused hearing loss at lower frequencies [37,38]. Hearing loss within the overall range of a frequency has also been described [27,28]. Several studies have shown that in workers exposed to noise, smoking may affect hearing loss at higher frequencies [13,15,16,21]. Although there are some exceptions, the current study showed that there was hearing loss within the overall range of a frequency. Considering that smoking may also affect the overall range of a frequency in the absence of exposure to noise, further studies are needed to examine the effects of smoking on hearing loss according to frequency in smokers who are exposed to noise.

In the current study, to evaluate the effects of the smoking amount on hearing function in current smokers who were exposed to noise, current smokers were divided into 4 groups according to pack-years, and then analyzed. We analyzed the data using the same method as that used for smoking status. The hearing threshold was highest within the overall range of frequencies other than 4 kHz in the ≥30 pack-years group. Multiple logistic regression analysis showed that at 1 k and 2 kHz, the severity of hearing loss was significantly high in all groups, except the 20–29.9 pack-years group at 1 kHz, as compared to the 0.05–9.9 pack-years group. There was no statistical significance at 3 k and 4 kHz. Some studies [2,14,39] have suggested that there is a significant correlation between the severity of hearing loss and response depending on the smoking amount. The current study obtained similar results at 1 k and 2 kHz as well. At 3 k and 4 kHz, however, these results could not be obtained. According to studies being conducted at present, smoking (1.0–3.3%) may affect sensorineural hearing loss but at a relatively lower degree as compared with age (26–48%) and noise (10–15%) [17]. In addition, it is also known that noise-induced hearing loss exhibits a maximal threshold shift at frequencies of approximately 3 k–6 kHz. It is therefore probable that the effects of long-term exposure to noise diluted that of smoking in the current study. It can therefore be inferred that there is a masking effect; thus, there would be no significant dose–response relationship at 3 k and 4 kHz. Indeed, some studies have reported that there is a dose–response relationship at higher frequencies in the absence of exposure to noise [35]. Although there were some exceptions in the current study, the severity of hearing loss was greater at lower frequencies if the workers who were exposed to noise were current smokers. In addition, our results also showed that a dose–response relationship existed. Although there was no dose–response relationship at higher frequencies, it is difficult to draw definite conclusions based solely on the current results. Further studies are therefore needed to support our results.

There are some limitations to the current study. First, because the current study was conducted using health examination data, serum levels of carboxyhemoglobin, RBC count, and hematocrit of only a few workers were measured. Therefore, we could not adjust these factors in multiple logistic regression analysis. As mentioned earlier, the effects of these factors cannot be overlooked when evaluating the effect of smoking on hearing loss. Unless these factors are adjusted, this would lower the statistical power. In addition, the issue of some data lacking consistency may arise. Second, it is probable that nicotine is absorbed into the body during smoking, and it may have a direct effect on the auditory organ. A mean comparison analysis of the levels of cotinine, one of the metabolic products synthesized from nicotine, would clarify the effects of nicotine [40-42]. This test was not performed in the current study. Third, while it was possible to control most of the confounding factors that might cause hearing loss, we could not control all factors, including past history of taking ototoxic drugs and workers who had congenital ear disorders. Presumably, this might have affected the current analysis. Finally, the current study was conducted using a cross-sectional design. Therefore, the temporal relationship is obscure and it was only possible to identify the correlation between smoking status and amount and the degree of hearing loss. However, it was difficult to identify a causal relationship between the 2 factors. Further well-designed prospective studies are needed to clarify the effects of smoking on the degree of hearing loss.

The current study is the first to demonstrate the correlation between smoking status and amount and the degree of hearing loss in Korean workers who are exposed to noise. Although there were limitations due to the cross-sectional design, we attempted to identify the correlation between smoking status and amount and the degree of hearing loss. There were significant results for some variables. Further long-term prospective studies are needed to overcome the limitations of the current study. This would probably yield more significant results on how smoking affects the degree of hearing loss in workers exposed to noise.

## Conclusion

In conclusion, our results showed that smoking might affect the degree of hearing loss within the overall range of a frequency in workers who are exposed to noise. It was also shown that heavy smoking had a greater effect on the degree of hearing loss at lower frequencies. At lower frequencies, there was a dose–response relationship based on the smoking amount. We could not confirm whether a dose–response relationship existed at higher frequencies. We could not draw conclusions based solely on our results. Although we could not confirm all the correlations, we could assume that smoking would have a detrimental effect on the degree of hearing loss in the workers exposed to noise by considering previous reports, the mechanisms by which smoking affects the degree of hearing loss, and our results. Therefore, of the factors that might affect noise-induced hearing loss, which has emerged as a serious problem in modern industrial society, smoking incurs enormous social expenditure and causes various cancers, respiratory diseases, cardiovascular diseases, and endocrine diseases. Moreover, it affects individuals indirectly. Proper management would reduce the occurrence of noise-induced hearing loss and prevent that of various diseases that stem from smoking. Thus, this strategy would contribute to improving the quality of life of workers and reducing social expenditure.

## Competing interests

The authors declare that they have no competing interests.

## Authors’ contributions

All authors had access to the data and played a role in writing this manuscript. CSS conceived and designed the study. JHS and CSS and JHL were involved in writing the manuscript. JHS and HL performed the data collection. CIY and CRL performed the statistical analysis, the interpretation of data. YHK had critically revised the manuscript. All authors read and approved the final manuscript.
